# Synthesis of In Situ ZrB_2_-SiC-ZrC Coating on ZrC-SiC Substrate by Reactive Plasma Spraying

**DOI:** 10.3390/ma15062217

**Published:** 2022-03-17

**Authors:** Bao-Xia Ma, Yang Wang, Si-Cong Zhao, Hao-Nan Wu, Yang Qiao

**Affiliations:** Department of Materials Science and Chemical Engineering, Harbin University of Science and Technology, Harbin 150040, China; wangyang2022wy@163.com (Y.W.); zscwr@163.com (S.-C.Z.); 18845042765@163.com (H.-N.W.); QY1043205196@163.com (Y.Q.)

**Keywords:** reactive plasma spraying (RPS), ZrB_2_-SiC, coating, ZrC, microstructure

## Abstract

In situ synthesis feasibility of ZrB_2_-SiC-ZrC composite coatings on ZrC ceramics by reactive plasma spraying (RPS) was investigated. To help to understand the phase evolution during plasma spraying process, reaction behavior in the ZrH_2_-Si-B_4_C system was explored carefully by differential scanning calorimetry. The results indicated that the phase transformation sequence in the ZrH_2_-Si-B_4_C system could be described as ZrH_1.66_, Zr_3_O, ZrC, ZrB_2_, Zr_2_Si, ZrSi, and SiC. The prior formation of ZrC was due to high diffusion rate of C atoms from B_4_C. ZrB_2_ was produced above 1100 °C. As the temperature increased, SiC were finally formed by the reaction of ZrC with ZrSi and B_4_C. The RPS composite coatings mainly consisted of ZrB_2_, SiC, and ZrC phases, except for a small fraction of ZrO_2_ phase. The microstructural characterization exhibited more dense melted splats, which appears to increase gradually with the increase in spraying currents and distances. The coatings had typical lamellar structure and adhered to the substrate well. The microhardness values were higher than 1000 HV_1_, but there were few variations with varying spraying currents and distances.

## 1. Introduction

Zirconium carbide (ZrC), which is known as a refractory and chemically stable compound, presents with a high melting point, high strength, high thermal conductivity, and chemical stability, and is a promising candidate for being a structural component used in next-generation rocket engines and hypersonic spacecrafts [1,2,3]. Unfortunately, poor high-temperature properties, such as thermal oxidation and ablation, prevent its use in many applications, including the aforementioned high-temperature structural applications. However, in most cases, the oxidation damage originates from the surface of the materials. Therefore, a coating is an effective solution to enhance the performance of ZrC substrate materials at extreme environments by surface modification.

ZrB_2_, with addition of 20–30 vol% SiC, exhibits good properties at high temperatures [4,5,6], which make it good candidate among coating materials applied on C/C composites [7,8]. Luckily, ZrB_2_-SiC [9,10] has a similar coefficient of thermal expansion (CTE) as ZrC [11], and has good chemical compatibility with ZrC [12]. These properties show that ZrB_2_-SiC ceramics are a feasible option for a protective coating of ZrC.

Among many fabrication methods, RPS combines atmospheric plasma spraying (APS) with self-propagation high-temperature synthesis (SHS), in which the desired phases for the coating could be formed in situ by the SHS reaction during the plasma spraying process [13]. It is suitable for the deposition of high temperature and refractory coatings [14,15,16].

The main objective of the present research was the feasibility of in situ synthesis ZrB_2_-SiC-ZrC composite coatings by RPS without transitional adhesive layer on ZrC ceramics. The coating was obtained by using an SHS reaction in ZrH_2_-Si-B_4_C mixed powders during the plasma jet. Presently, reports on reactive plasma sprayed coatings for ZrC ceramics substrate are relatively rare. Therefore, it is necessary to carry out basic work on the reaction behavior in the ZrH_2_-Si-B_4_C system and identify the fabrication characterizations of the coating. It is expected that the results obtained in this study can provide some valuable information for promoting the application of ZrC materials under ultra-high-temperature environments.

## 2. Experimental Methods

The raw powders used to prepare the ZrB_2_-SiC-ZrC coating were commercial powders of ZrH_2_ (>99% in purity, ≤38 μm), Si (>99% in purity, ≤5 μm), and B_4_C (>99% in purity, ≤3.5 μm). The mole ratio of ZrH_2_-Si-B_4_C powders was designed according to Equation (1) [17,18] to achieve ZrB_2_-based ceramics containing 20 vol% SiC.

xZrH_2_ + yB_4_C + (3y − x)Si → 2yZrB_2_ + (x − 2y)ZrC + (3y − x)SiC + xH_2_ (1)

**Figure 1:**
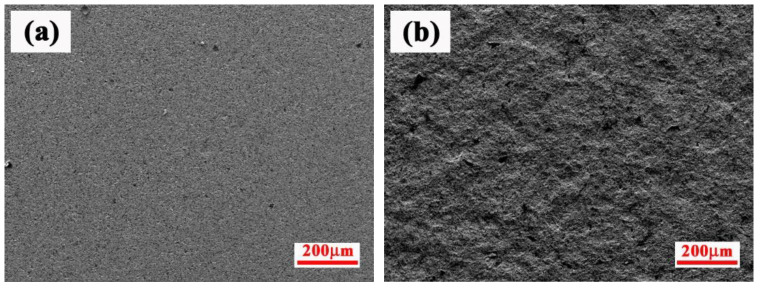
SEM morphology of substrate samples (**a**) before and (**b**) after grit-blasting.

**Figure 2:**
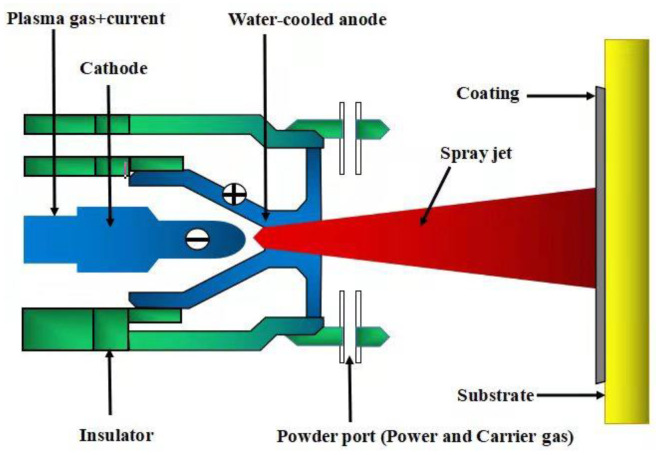
A schematic drawing of the plasma spray equipment.

The powder mixture was wet-mixed at 45 r/min for 36 h in a roller ball mill using ZrO_2_ balls with the additions of deionized water, Gum Arabic and tri-ammonium citrate, where Gum Arabic is a binder for agglomerating powders, and tri-ammonium citrate is a defoaming agent used to remove the bubbles in the mixture. The obtained slurry was spray-dried and then sieved to produce the agglomerated particles with 50–100 μm. The substrate samples of dimension 10 × 20 × 5 mm^3^ were cut from the ZrC-SiC composite billet. ZrC-SiC composites were prepared by hot pressed sintering and the details can be found in our previous work [19]. Before spraying, the substrate samples were grit-blasted by alumina sand and ultra-sonic-cleaned with ethanol. SEM morphology of the substrate before and after grit-blasting is shown in Figure 1. The ZrB_2_-SiC-ZrC coatings on the surface of the ZrC-SiC composites were prepared by the spraying apparatus, as schematically shown in Figure 2. The composite, agglomerated powders were deposited onto the substrate by atmospheric plasma spraying using a Unicoat Spraying System (F4 spraying gun, Sulzer Metco, Switzerland) with Ar and H_2_ as plasma gases, in which the flow rates of the gases were 35 L/min and 12 L/min, respectively. The flow rate of the carrier powder gas Ar was 2.5 L/min. Spray distances from the plasma gun nozzle to the substrate were selected as 80, 100, and 140 mm. These spraying parameters were based on previous work [20], and the detailed information is listed in Table 1. Each spraying experiment was performed about 3~5 times. Since ZrB_2_-SiC-ZrC coatings were first in situ synthesized by RPS on ZrC-SiC ceramic substrate, it is necessary to investigate the reaction process of the ZrH_2_-Si-B_4_C system. It should be noted that the reaction in the DSC apparatus was different from that in plasma spraying because of the disparity in the processing conditions, particularly for the heating rate and heat loss rate, but it was very similar, to some extent, as far as the reaction process. Therefore, the studies on DSC are valuable in understanding the reaction behavior and phase evolution during the plasma spraying process [21]. Thermal analysis of the composite agglomerated powders was carried out by an STA 449F3 Jupiter^®^-type thermal analyzer, and were conducted in a flowing argon gas using a heating rate of 10 °C/min. The phase constituents of DSC products and as-prepared coating were identified by X-ray diffraction (XRD, Philips X’-Pert PRO). The morphologies of DSC products, and the surfaces and cross-sections of the coatings, were characterized using a scanning electron microscope (SEM) (Model FEI Sirion-200, Holland) equipped with an energy-dispersive X-ray spectrometer (EDS). Furthermore, transmission electron microscopy (JEM-2100) was also employed to examine the surface microstructure of the coating. The hardness of the coatings was measured with a Vickers hardness tester (HV-1000A) at a load of 1000 g with a dwell time of 15 s (5 indents for each sample).

## 3. Results and Discussion

**Figure 3:**
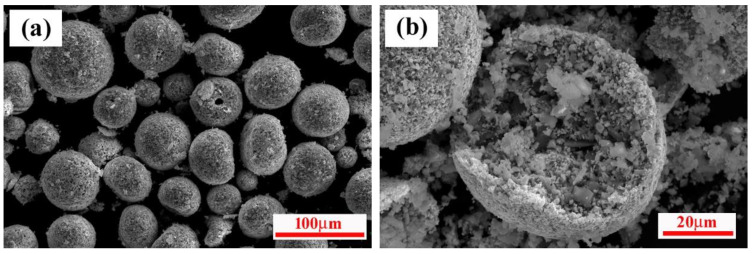
SEM micrographs of spray-dried agglomerated particles: (**a**) low magnification view and (**b**) fracture section morphology.

During the plasma spraying process, the size distribution and morphology of the composite agglomerated powders must be suitable, such as the uniform sphericity and proper cohesion strength, so as to ensure complete reaction in the plasma spraying process and to ensure uniform coatings [6,22]. The morphology of agglomerated particles used for RPS is shown in Figure 3. Spray-dried particles were spherical and near spherical, with sizes of 50–100 μm. The interior of the particles exhibited a relatively dense structure, implying good contact among the reactants. Meanwhile, every composite agglomerated particle can be recognized as an independent, SHS-reactive unit, which is beneficial to the ignition of the SHS reaction during the RPS process [14]. Thus, the characteristics of the composite powders endowed with better flowability of spray-dried particles and favored complete SHS reaction during the RPS process.

**Figure 4:**
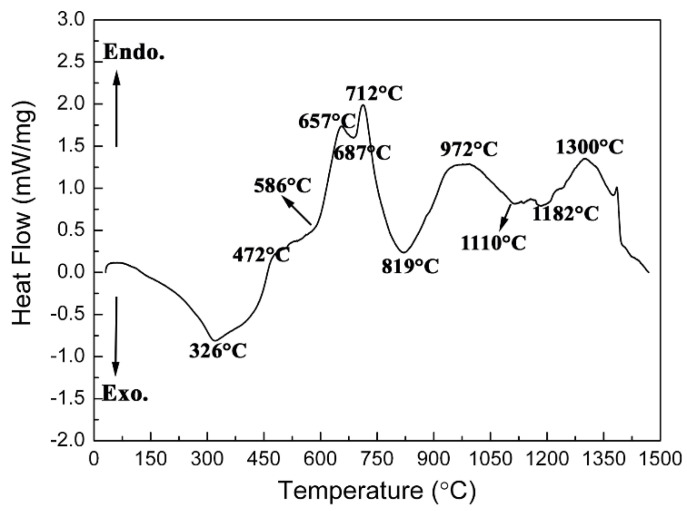
DSC curve for ZrH_2_-Si-B_4_C composite agglomerated powders.

**Figure 5:**
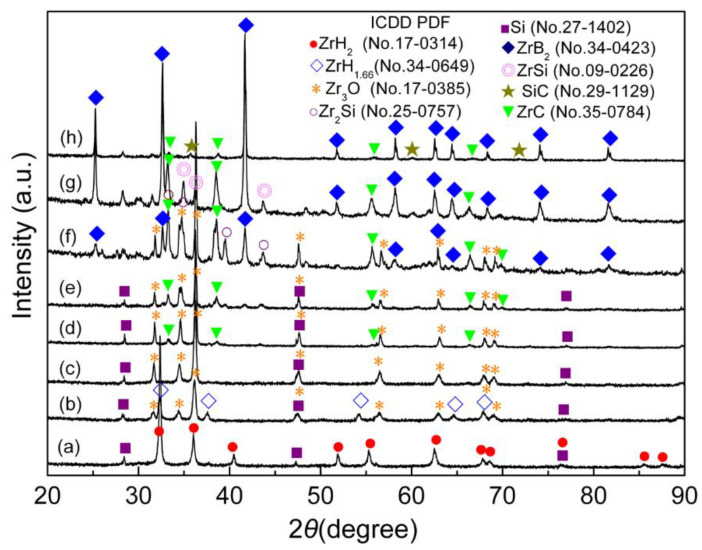
XRD diffractograms of the DSC products for ZrH_2_-Si-B_4_C composite agglomerated powders quenched at (**a**) 400 °C, (**b**) 700 °C, (**c**) 800 °C, (**d**) 960 °C, (**e**) 1100 °C, (**f**) 1200 °C, (**g**) 1300 °C, and (**h**) 1500 °C, respectively.

Figure 4 and Figure 5 show the DSC curve and the XRD diffractograms of DSC products at various temperatures for ZrH_2_-Si-B_4_C composite agglomerated powders, respectively. It can be found from Figure 4 that there were several continuous endothermic peaks below 700 °C. According to the XRD diffractograms of DSC products quenched at 400 °C and 700 °C, as shown in Figure 5a,b, it can be found that only ZrH_2_ and Si phases were detected below 400 °C; however, a large quantity of Zr_3_O and a trace of ZrH_1.66_ appeared, besides unreacted starting Si phase, when quenched at 700 °C, indicating that ZrH_2_ mainly decomposed between 400 °C and 700 °C. Additionally, the appearance of ZrH_1.66_ peaks implies that the dehydrogenation of ZrH_2_ was a gradual process with the sequence of ZrH_2_ and ZrH_x_, which is consistent with the result reported in Reference [17]. In this case, in the formation of the Zr_3_O phase, it can be presumed that ZrH_2_ powders or dehydrogenation products ZrH_x_ and Zr reacted with elemental O, which was derived from Gum Arabic and tri-ammonium citrate in the powder system. However, the provided amount of O in the system was not sufficient and the dehydrogenation of ZrH_2_ released hydrogen, which means that the system is in a reducing atmosphere, rich in H_2_, for a short time, thus favoring the formation of Zr_3_O instead of stable ZrO_2_. A strong exothermic peak appeared at about 819 °C. The corresponding XRD results heated to 800 °C in Figure 5c show that lots of Zr_3_O and unreacted Si were only detected in the final product. Therefore, it can be predicted that the conversion reaction of ZrH_x_ or Zr to Zr_3_O predominated at about 800 °C followed by continuous dehydrogenation of ZrH_x_. After a strong exothermic peak of 819 °C, an endothermic peak with the minimum at about 972 °C was observed in the DSC curve. The XRD result of the product heated to 960 °C in Figure 5d show that a trace of ZrC was found in addition to Zr_3_O and Si phases. Some researchers have concluded that B_4_C is primarily composed of B_11_C icosahedra, rich in boron, linked by C-B-C intericosahedral chains [23,24]. At high temperatures, carbon atoms are more easily dissociated compared with boron atoms; namely, the diffusivity of carbon from B_4_C crystal also is higher than that of boron [25]. In addition, because Zr is a more reactive metal than Si, any free Zr will preferentially react with C species to form Zr compounds and leave unreacted Si. Therefore, it can be concluded that C dissociated from B_4_C rapidly diffused into Zr_3_O, and reduced Zr_3_O to form ZrC, forming B-rich boron carbide at about 960 °C. In the temperature range of 1000 °C–1200 °C, several exothermic peaks were very close, which may be caused by continuous occurrence of several exothermic reactions. The corresponding quenched product, as shown in Figure 5e, suggests that the phase type at 1100 °C was similar to that at 960 °C, but the content of Zr_3_O significantly decreased and ZrC increased instead, illustrating that the transformation from Zr_3_O to ZrC was continuing. With quenching temperature rising above 1200 °C, two new phases of ZrB_2_ and Zr_2_Si were detected with the disappearance of Si phase, as shown in Figure 5f. Wu et al. [26] suggests that B and C atoms from B_4_C diffuse faster than Zr and Si. Additionally, Si has a lower reactivity than Zr, so ZrB_2_ and ZrC preferentially formed by reaction between Zr_3_O and B-rich or residual B_4_C. However, at this temperature, Si was also replaced by Si compounds. Similar to the Hf-Si system [27], the predominance of specific zirconium silicides at different temperatures logically depends on the activity ratio a_Zr_/a_Si_. At a high temperature of 1200 °C (compared with 1100 °C), Zr_2_Si may form as a result of a diffusion reaction between Zr_3_O and Si. However, at 1300 °C, Zr_2_Si transformed into ZrSi and disappeared at higher temperature of 1500 °C, which indicates that Zr_x_Si_y_ was unstable in the presence of B_4_C and Zr at high temperatures (above 1300 °C). In addition, the peak of Zr_3_O completely disappeared, and ZrB_2_ became the main phase at temperatures from 1300 °C to 1500 °C. Additionally, SiC appeared with a disappearance of Zr_x_Si_y_ peaks and a decrease in the intensities of ZrC peaks, based on the XRD diffractograms. This phenomenon supports the conclusion that the formation of SiC derived from the reaction between ZrC, ZrSi, and residual B_4_C in this temperature region.

**Figure 6:**
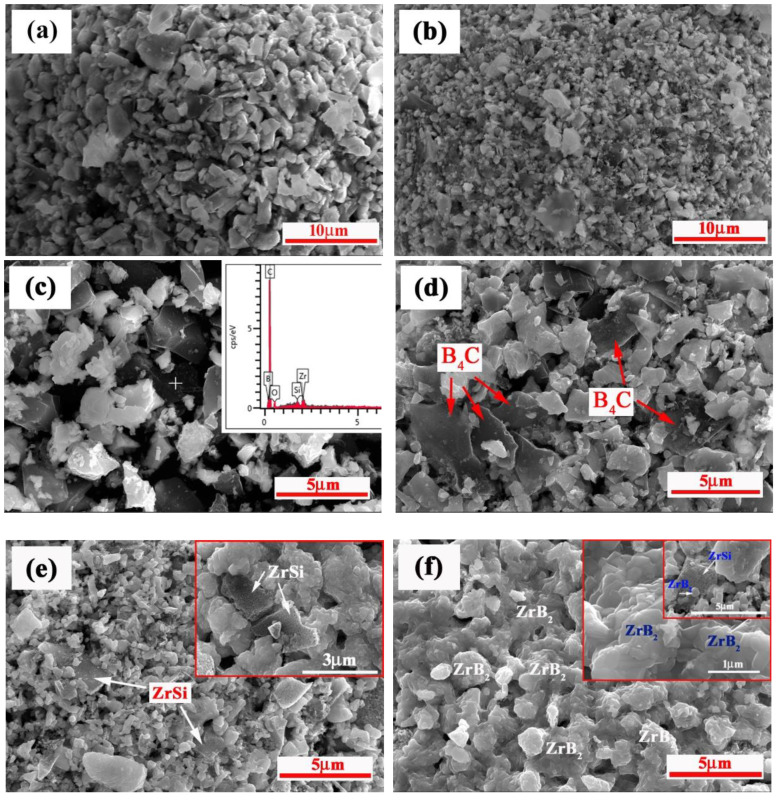
SEM images of the DSC products for ZrH_2_-Si-B_4_C composite agglomerated powders at (**a**) room temperature, and quenched at (**b**) 800 °C, (**c**) 960 °C, (**d**) 1200 °C, (**e**) 1300 °C, and (**f**) 1500 °C, respectively.

The typical SEM images of the raw powder and the products at various temperatures of 800 °C, 960 °C, 1200 °C, 1300 °C, and 1500 °C are shown in Figure 6a–f, respectively. As indicated, the surface of agglomerated particles quenched at 800 °C seemed to be denser and finer compared with that of spray-dried particles. The reasons may be one of the following: one is that the particle size of ZrH_2_ was further decreased due to its decomposition into ZrH_1.66_ above 700 °C (as shown in Figure 5 b,c); the other is that, during ball mixing, some Si particles were in high-energy state and had a higher reactivity, except for the size reduction, which causes the small-sized Si with the low melting point to melt slightly first, and fill the pores between the particles. Figure 6c shows the internal microstructures of agglomerated particles at 960 °C; it can be observed that the components kept the original irregular block shape. The EDS embedded in Figure 6c revealed that the surface of B_4_C was rich in a considerable amount of C (80.71 at.%, which is higher than 32.42 at.% of C in raw B_4_C particle), which agrees with the results in Reference [28]. The study suggested that the carbon photoabsorption spectrum of B_4_C changed when the temperatures were higher than 1000 K. At this moment, the surface of B_4_C was covered with a graphite layer, leaving B-rich boron carbide buried beneath the graphite surface. The above findings and suggestions support the results obtained in Figure 6c of this study, e.g., the rapid diffusion of carbon from B_4_C and the prior formation of ZrC. Figure 6d shows typical microstructure of the products at 1200 °C. As shown, with the temperature increasing, many scaly ZrB_2_ particles were found on the surface of light gray compound particles containing Zr. Additionally, the surface of dark gray B_4_C particles became rough, suggesting that B and C atoms form B_4_C diffused out to react and were consumed. Figure 6e,f show typical microstructures of the products at 1300 °C and 1500 °C, respectively. Clearly, some partial or complete melts were more visible. Additionally, a large amount of of ZrB_2_ particles were observed in the products, especially at 1500 °C, ZrB_2_ particles grew into obvious hexagonal shape. Additionally, large grey bulk phase in Figure 6f was identified as ZrSi, and ZrB_2_ was traced to grow from ZrSi particles (as shown in the image embedded in Figure 6f). All microstructures observations are consistent with the XRD identification results. From the above results, the possible reaction process of system during plasma spraying could be inferred, which are shown in Equations (2)–(8), as follows:

ZrH_2_→ZrH_1.66_ + 0.17H_2_ (2)

3ZrH_1.66_ + [O]→Zr_3_O + 2.49H_2_ (3)

Zr_3_O + 4[C]→3ZrC + CO (4)

3Zr_3_O + 4B_4_C→8ZrB_2_ +ZrC + 3CO (5)

2Zr_3_O + 5Si→3Zr_2_Si + 2SiO (6)

B_4_C + 3Zr_2_Si → 2ZrB_2_ + ZrC + 3ZrSi (7)

3ZrSi + 2B_4_C + ZrC→3SiC + 4ZrB_2_ (8)

**Figure 7:**
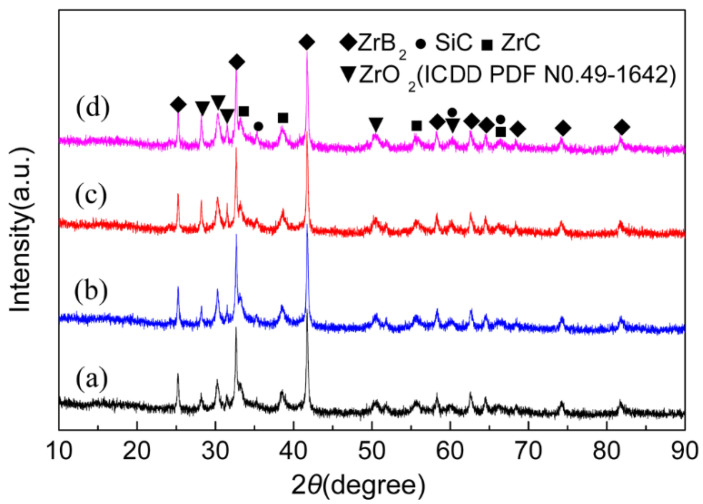
XRD diffraction patterns of ZrB_2_-SiC-ZrC coatings prepared by RPS: (**a**) 550A, 80 mm, (**b**) 600A, 80 mm, (**c**) 600A, 100 mm, and (**d**) 600A, 140 mm.

**Figure 8:**
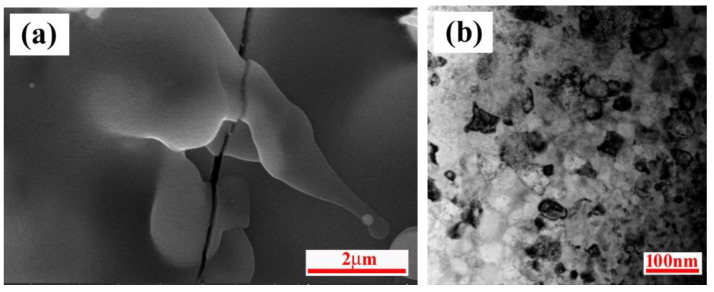
(**a**) SEM and (**b**) TEM micrographs of ZrB_2_-SiC-ZrC coating.

The information obtained from the DSC experiments is not only helpful for the understanding of reaction process during plasma spraying, but also provides a fact that the objective product of ZrB_2_-SiC-ZrC can be synthesized by the ZrH_2_-Si-B_4_C system. The X-ray diffraction patterns of the coatings prepared by RPS using ZrH_2_-Si-B_4_C system are shown in Figure 7. Similar to the DSC results, ZrB_2_, SiC, and ZrC phases existed in the coating, while a small amount of ZrO_2_ remained, and no other unreacted or intermediate phases were found. It is considered that the reactions in RPS were almost complete according to Equation (1). In this case, the formation of ZrO_2_ was inevitable, since the RPS process was performed in air, and the air was also involved under the disturbing effect of the plasma jet on its surrounding air. Consequently, oxidation behavior occurred through the chemical reactions between the liquid droplet and oxygen during the coating process or after deposition onto the substrate. Besides, the change of ZrO_2_ peak intensity with increasing the spray distance was more noticeable than that with increasing arc current, which may be attributed to the longer dwell time in the plasma jet. In addition, the relative intensity of SiC was low in the coating. On the basis of previous works [4,29], SiC can decompose during spraying, which can be reduced by a eutectic phase formed between ZrB_2_ and SiC. In the present experimental work, the synthesis of ZrB_2_-SiC-ZrC coating by ZrH_2_-Si-B_4_C powder was carried out under the combined effects of plasma jet and reaction heat release of ZrH_2_-Si-B_4_C powder, where the higher system temperature may have facilitated the decomposition of SiC. Additionally, a possible formation of a ZrB_2_-SiC mixture was beneficial to prevent the decomposition. However, it should be noted that eutectic-like regions have not been found, this hypothesis still needs to be verified. It is also noticeable from Figure 7 that the diffraction peaks of the coating were low and broad, which means the phases in the coating had lower crystallinity and smaller grain size due to extremely high cooling rate. This presumption is in accordance with their high magnification SEM and TEM pictures, which are displayed in Figure 8. The SEM image (Figure 8a) reveals that the splats in the reactive plasma sprayed ZrB_2_-SiC-ZrC coating seems to be amorphous melt, and TEM image (Figure 8b) indicates that the coatings were composed of a large number of nanosized grains.

**Figure 9:**
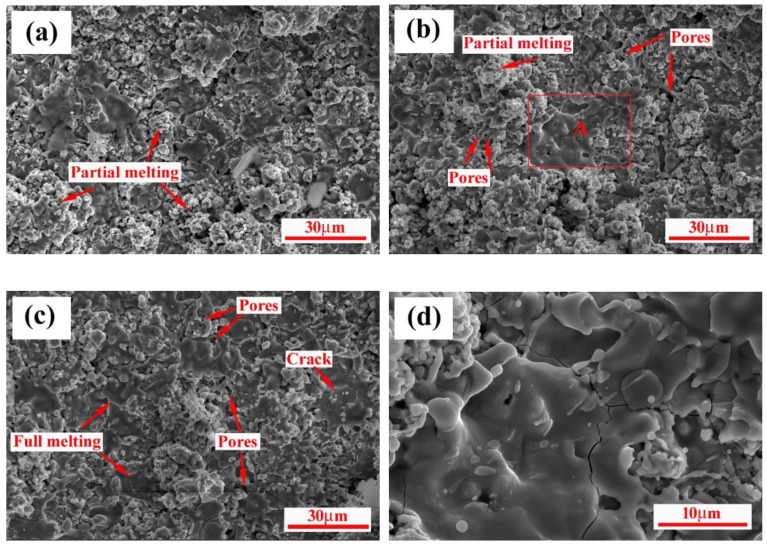
Surface morphologies of ZrB_2_-SiC-ZrC coatings prepared by RPS under different spraying parameters: (**a**) 550A, 80 mm, (**b**) 600A, 100 mm, (**c**) 600A, 140 mm, and (**d**) high magnification of A zone in (**b**).

Figure 9 is the surface SEM images of the coating. The desirable surface morphology of the ZrB_2_-SiC-ZrC coating, which has no obvious macro defects or cracks, was accomplished by utilizing a similar CTE principle between the substrate and the coating to minimize the effect of the mismatched CTE. The microstructural characterization of all of the coatings demonstrates that, except for a few large stacking regions which are composed of insufficiently molten powders, more dense, disk-like or pancake splats consisting of fully molten regions were found in the sprayed coating, and formed a flattened, smooth area. This splat pattern gave rise to coatings endowed with higher adhesive and cohesive strength [6,30]. It should be noted that the splats increase gradually with the increase in spraying currents and distances. This might be related with an increase in particle temperature, since an increase in spraying current increases the heat energy of the plasma jet and an increase in spraying distances causes the longer dwell time in plasma jet [31]. Additionally, a few splash structures were also observed, and the morphology of solidifying droplets was formed, as depicted in Figure 9d. This splash structure resulted from the fact that in-flight particles, on impact with the substrate, fragmented into smaller multiple droplets, and then solidified rapidly. Additionally, some microcracks and pores were distributed on surface of the coating. The formation of the microcracks may be attributed to be the shrinkage stress caused by rapid cooling of the coating. Incomplete overlapping between the splats might result in the appearance of some pores.

**Figure 10:**
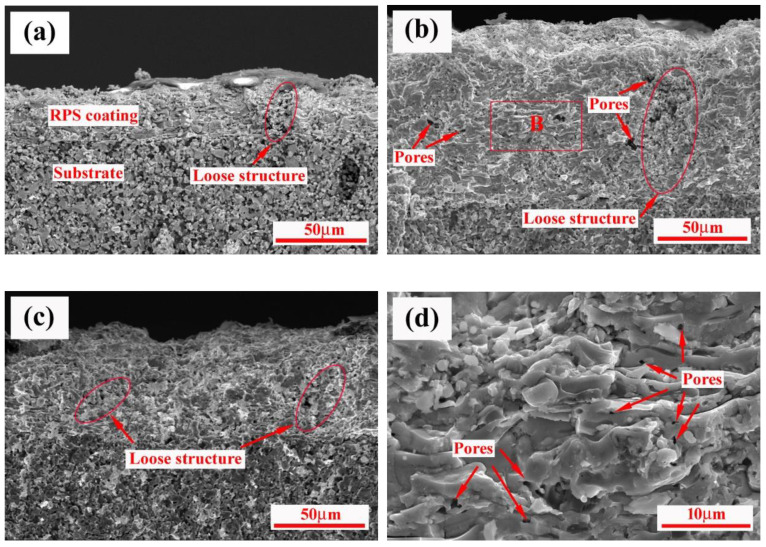
Cross-sectional micrographs of the RPS ZrB_2_-SiC-ZrC coatings: (**a**) 550A, 80 mm, (**b**) 600A, 80 mm, (**c**) 600A, 100 mm, and (**d**) high magnification of B zone in (**b**).

Figure 10 shows the cross-section of the coating samples observed by SEM. It can be seen that the coating shows uniform thickness (in the range of 250–300 μm) and good interfacial adhesion. It can be seen that internal microstructure of the coating consists of two different regions which are easily distinguishable: loose and high pore region and compact lamellar region. In high magnification of Figure 10d, the intimate lamella is more noticeable; however, a small number of micropores are also clearly visible. The reason for the formation of the micropores may be explained: the molten droplets were deposited onto the substrate and splashed to form the shadowing effect, resulting in the enwrapped gas not being expelled promptly.

As is well known, RPS is a process that utilizes the heat of high-temperature plasma to initiate combustion synthesis reactions [32]. In this process, once the exothermic reactions are ignited, self-propagating proceeds quickly, and the temperature of the system increases significantly in a short time. The powders, entering the plasma jet, will form a molten state, will convert the reactant particles into final products during in-flight processes, and then will impinge on the substrates as splats. With time, the continuous deposition of splats results in the formation of dense lamella structures. However, in fact, the powders appear to be emanative after they are ejected from the plasma nozzle, and thus some of the powders may be scattered outside the plasma jet and will form a semi-molten state to complete the synthesis reaction during spraying, and they subsequently impact on the substrates as a loose, stacked structure. The phenomenon will be strengthened if large-sized powder particles are involved, outside of the plasma jet. The microstructure characteristic in Figure 10 supports the results of the surface observation.

**Figure 11:**
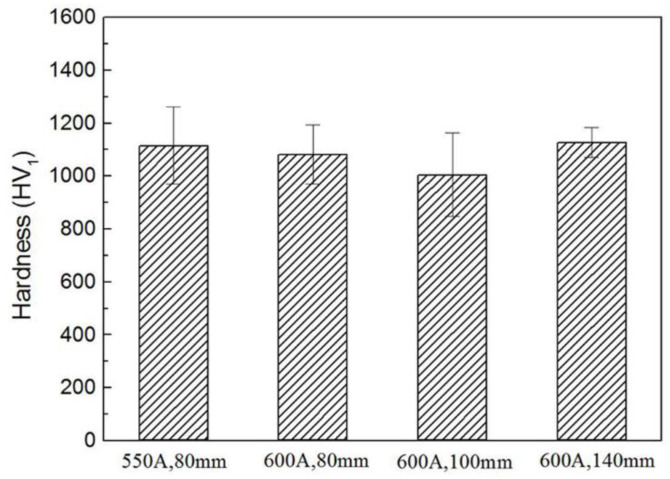
Microhardness of the ZrB_2_-SiC-ZrC coatings.

Figure 11 illustrates the microhardness results of ZrB_2_-SiC-ZrC coatings. All the microhardness values of the coatings are higher than 1000 HV_1_, although no obvious variation with varying spraying currents and distances can be observed, and are statistically identical to each other, considering the standard deviation of the data. The attainment of high hardness is presumably due to the more compact structure of the coating deposited by RPS and the high hardness of the hard phases, such as ZrB_2_, SiC, and ZrC.

## 4. Conclusions

Dense and well-bonded ZrB_2-_SiC-ZrC composite coatings were successfully prepared in situ by reactive plasma sprayed ZrH_2_-Si-B_4_C powders. Reaction behavior in the ZrH_2_-Si-B_4_C system was investigated through phase analysis and microstructures of quenched samples by differential scanning calorimetry. The phase transformation sequence in the ZrH_2_-Si-B_4_C system could be described as ZrH_1.66_ and Zr_3_O at 700 °C, ZrC at 960 °C, ZrB_2_ and Zr_2_Si at 1200 °C, ZrSi at 1300 °C, and SiC at 1500 °C. The prior formation of ZrC over ZrB_2_ was due to the fact that the diffusion rate of C atoms was higher than that of B atoms in B_4_C. As the temperature increased, SiC were finally formed by the reaction of ZrC with ZrSi and B_4_C. The phase composition of RPS coating consisted of ZrB_2_, SiC, ZrC, and a small quantity of ZrO_2_. Microstructure characterization exhibited more dense disk-like or pancake splats, except for a few large stacking structures. By utilizing a similar CTE principle between the substrate and the coating, the absence of macro defects and cracks, and good interfacial bonding, were obtained. In contrast, the microhardness value of the coating exhibited few variations with various spraying currents and distances. In this work, all obtained results indicated that it was feasible to fabricate ZrB_2_-SiC-ZrC ceramics coatings, without an adhesive layer, on ZrC ceramic substrate by the RPS method. For high-temperature application coatings, research on the mechanical behaviors and the failure mechanisms of ZrB_2-_SiC-ZrC coatings in high-temperature environments must urgently be carried out in future work.

## Figures and Tables

**Table 1:** Reactive plasma spraying parameters of the ZrB_2_-SiC-ZrC coating.

Sample Number	Spraying Current (A)	Spraying Voltage (V)	Primary Gas Ar (L/min)	Second Gas H_2_ (L/min)	Carrier Gas Ar (L/min)	Spraying Distance (mm)
1	550	64	35	12	2.5	80
2	600	64	35	12	2.5	80
3	600	64	35	12	2.5	100
4	600	64	35	12	2.5	140

## Data Availability

Data is contained within the article.

## References

[1] Opeka M.M., Talmy I.G., Wuchina E.J., Zaykoskia J.A., Causeyb S.J. (1999). Mechanical, Thermal, and Oxidation Properties of Refractory Hafnium and Zirconium Compounds. J. Eur. Ceram. Soc..

[2] Ogawa T., Ikawa K. (1981). High-temperature Heating Experiments on Unirradiated ZrC Coated Fuel Particles. J. Nucl. Phys. Mater. Sci. Radiat. Appl..

[3] Feng L., Fahrenholtz W.G., Hilmas G.E. (2019). Effect of ZrB_2_ Content on the Densification, Microstructure, and Mechanical Properties of ZrC-SiC Ceramics. J. Eur. Ceram. Soc..

[4] Tului M., Marino G., Valente T. (2006). Plasma Spray Deposition of Ultra High Temperature Ceramics. Surf. Coat. Technol..

[5] Monteverde F., Savino R. (2007). Stability of Ultra-high-temperature ZrB_2_-SiC Ceramics under Simulated Atmospheric Re-entry Conditions. J. Eur. Ceram. Soc..

[6] Bartuli C., Valente T., Tului M. (2002). Plasma Spray Deposition and High Temperature Characterization of ZrB_2_-SiC Protective Coatings. Surf. Coat. Technol..

[7] Yao X.Y., Li H.J., Zhang Y.L., Li K.Z., Fu Q.G., Peng H. (2013). Ablation Behavior of ZrB_2_-based Coating Prepared by Supersonic Plasma Spraying for SiC-coated C/C Composites under Oxyacetylene Torch. J. Therm. Spray Technol..

[8] Zhang Y.L., Hu Z.X., Yang B.X., Ren J.C., Li H.J. (2015). Effect of Pre-oxidation on the Ablation Resistance of ZrB_2_-SiC Coating for SiC-coated Carbon/Carbon Composites. Ceram. Int..

[9] Alosime E.M., Alsuhybani M.S., Almeataq M.S. (2021). The Oxidation Behavior of ZrB_2_-SiC Ceramic Composites Fabricated by Plasma Spray Process. Materials.

[10] Mallik M., Kailath A.J., Ray K.K., Mitraa R. (2012). Electrical and Thermophysical Properties of ZrB_2_ and HfB_2_ Based Composites. J. Eur. Ceram. Soc..

[11] Wu H., Li H.J., Fu Q.G., Yao D.J., Wang Y.J., Chao M., Wei J.F., Han Z.H. (2011). Microstructures and Ablation Resistance of ZrC Coating for SiC-coated Carbon/Carbon Composites Prepared by Supersonic Plasma Spraying. J. Therm. Spray Technol..

[12] Kim K.H., Shim K.B. (2003). The Effect of Lanthanum on the Fabrication of ZrB_2_-ZrC Composites by Spark Plasma Sintering. Mater. Character.

[13] Yan D.R., Dai X.R., Yang Y., Dong Y.C., Chen X.G., Chu Z.H., Zhang J.X. (2018). Microstructure and Properties of in-situ Ceramic Matrix Eutectic Nanocomposite Coating Prepared by Plasma Spraying Al-Cr_2_O_3_-Al_2_O_3_ powder. J. Alloys Compd..

[14] Xu J.Y., Zou B.L., Fan X.Z., Zhao S.M., Hui Y., Wang Y., Zhao X., Cai X.L., Tao S.Y., Ma H.M. (2014). Reactive Plasma Spraying Synthesis and Characterization of TiB_2_-TiC-Al_2_O_3_/Al Composite Coatings on a Magnesium Alloy. J. Alloys Compd..

[15] Kim J.M., Lee S.G., Park J.S., Kim H.G. (2014). Laser Surface Modification of Ti and TiC Coatings on Magnesium Alloy. Phys. Met. Metallogr..

[16] Liu H.Y., Huang J.H., Yin C.F., Zhang J.G., Lin G.B. (2007). Microstructure and Properties of TiC-Fe Cermet Coatings by Reactive Flame Spraying Using Asphalt as Carbonaceous Precursor. Ceram. Int..

[17] Ran S.L., Biest O.V., Vleugels J. (2010). ZrB_2_-SiC Composites Prepared by Reactive Pulsed Electric Current Sintering. J. Eur. Ceram. Soc..

[18] Qu Q., Han J.C., Han W.B., Zhang X.H., Hong C.Q. (2008). In Situ Synthesis Mechanism and Characterization of ZrB_2_-ZrC-SiC Ultra High-temperature Ceramics. Mater. Chem. Phys..

[19] Ma B.X., Han W.B., Guo E.J. (2014). Oxidation Behavior of ZrC-based Composites in Static Laboratory Air up to 1300 °C. Int. J. Refract. Met. Hard Mater..

[20] Ma B.X., Li J.Y. (2019). ZrB2-SiC-ZrC Coating on ZrC Ceramics Deposited by Plasma Spraying. Results Phys..

[21] Liang Y.H., Han Z.Z., Lin Z.H., Ren L.Q. (2012). Study on the Reaction Behavior of Self-propagating High-temperature Synthesis of TiC Ceramic in the Cu-Ti-C System. Int. J. Refract. Met. Hard Mater..

[22] Singh V., Diaz R., Balani K., Agarwal A., Seal S. (2009). Chromium Carbide-CNT Nanocomposites with Enhanced Mechanical Properties. Acta. Mater..

[23] Kwei G.H., Morosin B. (1996). Structures of the Boron-rich Boron Carbides from Neutron Powder Diffraction: Implications for the Nature of the Inter-icosahedral Chains. J. Phys. Chem..

[24] Lazzari R., Vast N., Besson J.M., Baroni S., Corso A.D. (1999). Atomic Structure and Vibrational Properties of Icosahedral B4C Boron Carbide. Phys. Rev. Lett..

[25] Shen P., Zou B.L., Jin S.B., Jiang Q.C. (2007). Reaction Mechanism in Self-propagating High Temperature Ssynthesis of TiC-TiB_2_/Al Composites From an Al-Ti-B_4_C System. Mater. Sci. Eng. A.

[26] Wu W.W., Zhang G.J., Kan Y.M., Wang P.L. (2006). Reactive Hot Pressing of ZrB_2_-SiC-ZrC Ultra High-temperature Ceramics at 1800 °C. J. Am. Ceram. Soc..

[27] Monteverde F. (2005). Progress in the Fabrication of Ultra-high-temperature Ceramics: “In Situ” Synthesis, Microstructure and Properties of a Reactive Hot-pressed HfB_2_-SiC Composite. Compos. Sci. Technol..

[28] Jimenez I., Sutherland D.G.J., van Buuren T., Carlisle J.A., Terminello L.J., Himpsel F.J. (1998). Photoemission and X-ray-absorption Study of Boron Carbide and Its Surface Thermal Stability. Phys. Rev. B.

[29] Tului M., Giambi B., Lionetti S., Pulci G., Sarasini F., Valente T. (2012). Silicon Carbide Based Plasma Sprayed Coatings. Surf. Coat. Technol..

[30] Bianchi L., Leger A.C., Vardelle M., Vardelle A., Fauchais P. (1997). Splat Formation and Cooling of Plasma-sprayed Zirconia. Thin Solid Films.

[31] Baik K.H., Seok H.K., Kim H.S., Grant P.S. (2005). Non-equilibrium Microstructure and Thermal Stability of Plasma-sprayed Al-Si Coatings. J. Mater. Res..

[32] Deevi S.C., Sikka V.K., Swindeman C.J., Seals R.D. (1997). Application of Reaction Synthesis Principles to Thermal Spray Coatings. J. Mater. Sci..

